# Exosomes in multiple sclerosis and Alzheimer's disease – Adversary and ally

**DOI:** 10.1016/j.bj.2023.100665

**Published:** 2023-09-29

**Authors:** Norina Tang

**Affiliations:** a Department of Periodontics, University of the Pacific, San Francisco, USA; b Department of Laboratory Medicine, San Francisco Veterans Affairs Health Care System, San Francisco, USA

**Keywords:** Exosomes, Extracellular vesicles, Alzheimer's disease, Multiple sclerosis, Biomarkers

## Abstract

Neuroinflammation and the resulting neurodegeneration is a big challenge for the healthcare system, especially with the aging population. Neuroinflammation can result from a variety of insults to the central nervous system leading to an interplay between immune and brain cells that sustains chronic inflammation and injures neural cells. One facilitator of this toxic interplay are exosomes. Exosomes are nano-sized, bilayer lipid vesicles secreted by cells containing proteins, nucleic acids and lipids. Because exosomes can be internalized by other cells, their contents can elicit inflammatory responses and trigger toxicities in recipient cells. On the flip side, exosomes can act as therapeutic vehicles carrying protective cargo to maintain homeostasis. This review discusses exosome biogenesis, composition, and its role in neuroinflammation and neurodegeneration in the context of multiple sclerosis and Alzheimer's disease. The emerging roles of exosomes as biomarkers of neurologic diseases and as therapeutic delivery vehicles are also discussed. With all of these varying roles, interest and excitement in exosomes continue to grow exponentially and their promise as brain therapeutics is only beginning to be explored and harnessed.

## Highlights

• Exosomes are nano-sized, bilayer lipid vesicles secreted by cells containing proteins, nucleic acids and lipids.
• Exosomes can elicit inflammation and toxicity, or act as therapeutic vehicles carrying protective cargo for homeostasis.
• As a form of liquid biopsy, exosomes can serve as biomarkers of multiple sclerosis and Alzheimer's disease.
• Exosomes can be engineered and exploited to serve as therapeutic delivery vehicles for brain diseases.

## Introduction

Neuroinflammation and the associated neurodegeneration is a big challenge for the healthcare system, especially with the aging world population. Globally, neurological diseases are the leading cause of disability and the second leading cause of mortality. It is often the case that neuroinflammation and neurodegeneration go hand-in-hand in many central nervous system (CNS) disorders. In some CNS diseases such as multiple sclerosis (MS), spinal cord injury, traumatic brain injury and stroke, inflammation is primary which then leads to secondary neurodegeneration. In other CNS diseases such as Alzheimer's disease (AD), Parkinson's disease, amyotrophic lateral sclerosis and Huntington's disease, neurodegeneration is primary, leading to secondary reactive inflammation. The prototypical CNS disease example of the former is MS while AD is the most common example of the latter.

Globally, MS affects approximately 2.8 million people as of the year 2020 [1] while AD affects roughly 40 million people as of 2019 [2]. Although there are phenotypic and neuropathologic differences between MS and AD, research studies to date suggest they are similar in that both are driven by the interplay between genetic and environmental factors that culminate in disruptions to the immune system and CNS. Many risk factors have been described for each of these diseases; however, the exact cause for each is unknown. In addition to the many genetic and environmental factors associated with these multifactorial, neurodegenerative diseases, extracellular vesicles (EVs) have recently been implicated.

EVs are small, membrane-enclosed particles secreted by cells under normal as well as pathological conditions. EVs are devoid of a nucleus and cannot replicate. EVs carry proteins, lipids and nucleic acids which reflect the state of the parental cells secreting them and when internalized, their cargo can affect the function of recipient cells. There are three major classes of EVs that are differentiated based on their method of biogenesis: microvesicles, exosomes and apoptotic bodies. Of the three, exosomes are the most widely studied. This review focuses on the role exosomes play in modulating neuroinflammation and neurodegeneration in the pathogenesis of MS and AD. In addition, the role exosomes play as potential biomarkers and therapeutics of these neurodegenerative diseases is discussed.

## Exosome biogenesis

Almost all cells, both eukaryotic and prokaryotic, release EVs. EVs were first observed in 1946 as procoagulant platelet-derived particles in normal plasma. Since its discovery, EVs have been isolated from a variety of biological fluids, including blood, urine, tears, saliva, breast milk, bronchoalveolar lavage, amniotic fluid, synovial fluid, ascites, cerebrospinal fluid, bile and semen [3]. EVs were initially simply thought of as “garbage cans” into which unwanted materials were thrown into, that are then released from the cells. However, evidence in the past decade indicates that EVs are signaling vessels delivering cell-specific cargos of proteins, lipids and nucleic acids to other cells where they can alter function and physiology.

**Fig. 1:**
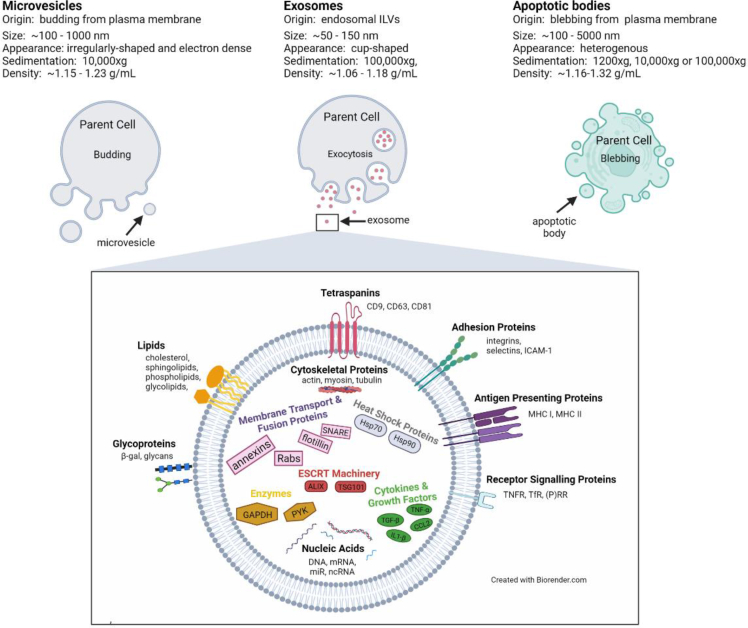
**Extracellular vesicle (EV) classes, origins and characteristics.** The three major classes of EVs are depicted based on their origins. Microvesicles and apoptotic bodies are generated via budding and blebbing of the plasma membrane, respectively. Exosomes are generated via the endocytic pathway by exocytosis of the intraluminal vesicles of multivesicular bodies that fuse with the plasma membrane. EV size [4], appearance by electron microscopy [85], sedimentation [85], and density [4] are shown. Proteins, lipids and nucleic acids frequently found in exosomes, which largely reflect their endocytic origin, along with some cell-specific molecules are illustrated. Abbreviations: ALIX: ALG-2-interacting protein X; β-gal:β-galactosidase;CCL2:chemokine (C–C motif) ligand 2;GAPDH:glyceraldehyde-3-phosphate dehydrogenase;Hsp:heat shock protein; ICAM-1:intercellular adhesion molecule 1; IL-1β:interleukin 1β; MHC:major histocompatibility complex; miR:microRNA; ncRNA:non-coding RNA; (P)RR:(pro)renin receptor; PYK:pyruvate kinase; Rabs:Ras-associated binding proteins; SNARE:soluble N-ethylmaleimide-sensitive fusion attachment protein receptor; TfR:transferrin receptor; TGF-β: transforming growth factor β; TNF-α: tumor necrosis factor α; TNFR: tumor necrosis factor receptor; TSG101: tumor susceptibility gene 101.

EVs can be generally divided into three main classes depending on their origins: apoptotic bodies, microvesicles and exosomes [4]. Apoptotic bodies are blebs from the plasma membrane of apoptotic cells with sizes ranging from ∼100 to 5000 nm. Microvesicles are smaller, ∼100–1000 nm, and bud directly from the plasma membrane of living cells. Exosomes are the smallest of the three, at ∼50–150 nm in size. In contrast to the other two EV classes, exosomes are formed by the inward invagination of endosomal membranes, giving rise to multivesicular bodies (MVBs) that either fuse with lysosomes or fuse with the plasma membrane. When fused with the plasma membrane, MVBs release their intraluminal vesicles (ILVs) into the extracellular milieu via exocytosis where they then are called exosomes [Fig. 1].

## Exosome composition

During its formation, the multivesicular endosomes take up bits of the cytoplasm and its contents, becoming part of the exosome. Thus, exosomal cargo reflects the identity and real time physiologic state of the parental secreting cell. There is no one single marker that can uniquely identify exosomes or other subpopulations of EVs. Nevertheless, specific proteins have been identified that are enriched in exosomes that reflect their biogenesis [Fig. 1]. These include tetraspanin proteins (CD9, CD63, and CD81) present in the endosomal membrane, endosomal sorting complex required for transport (ESCRT) proteins (ALIX and TSG101) that regulate cargo targeting into and the formation of ILVs, proteins required for membrane transport and fusion (annexins, Rabs, flotillins, SNAREs), heat shock proteins (Hsp70, Hsp90) that help recruit cytosolic components, membrane lipids (cholesterol, sphingomyelin, ceramides, glycerophospholipids), cytoskeletal proteins (actin, tubulin, cofilin), major histocompatibility complex (MHC) I proteins, integrins, and mitochondrial proteins [3]. A variety of glycoproteins as well as cell type-specific exosomal proteins, such as MHC II molecules from antigen presenting cells, have also been described [3,5]. In addition to proteins and lipids, exosomes also contain nucleic acids: DNA, mRNA, microRNA (miR), non-coding RNA (ncRNA) and other small RNA species [3]. Small ncRNAs ranging in length from 4 to 40 nucleotides, such as miRs, are particularly enriched, with exosomal miRs having a different profile than parental cells due to active sorting [3]. [Table 1] lists examples of molecules reported in exosomes. There are also public online databases such as ExoCarta (www.exocarta.org), vesiclepedia (www.microvesicles.org) and EVpedia (www.evpedia.info) that are dynamically cataloging the many different components found in exosomes and other EVs.

**Table 1:** Exosome composition.

Class	Examples
*Proteins*	
Tetraspanins	CD9, CD63, CD81
ESCRT machinery	ALIX, TSG-101
Membrane transport and fusion	annexins, flotillins, Rabs, SNAREs
Heat shock	Hsp70, Hsp90
Cytoskeleton	actin, cofilin, tubulin
Mitochondrial proteins	humanin, citrate synthase
Major histocompatibility complex	MHC I, MHC II
Adhesion	ICAM-1, integrin-α, -β, P-selectin
Glycoproteins	β-gal, glycans
Growth factors and cytokines	CCL2, FasL, IL-1β, TGF-β, TNF-α, TRAIL
Receptors	EGFR, TfR, TNFR
*Nucleic Acids*	
DNA	*Braf*, *Egfr*, mtDNA
mRNA	*Icam-1, Il-6*, *Tnf*
miR	miR-150, miR-320, miR-451
ncRNA	LncRNA, rRNA, tRNA, snRNA
*Lipids*	
Steroids	cholesterol, sex hormones
Sphingolipids	ceramides, sphingomyelin, gangliosides
Glycerophospholipids	PS, PC, PE, PI

Abbreviations: *Braf:*b-raf protooncogene; *Egfr*:epidermal growth factor; FasL:Fas ligand; LncRNA:long non-coding RNA; mtDNA:mitochondrial DNA; PC:phosphatidylcholine; PE:phosphatidylethanolamine; PI:phosphatidylinositol;PS: phosphatidylserine; snRNA:small nuclear RNA; TRAIL:TNF-related apoptosis inducing ligand.

## Exosome sample purity and nomenclature

Because EVs are heterogeneous in size and composition with physical characteristics that overlap with other particles, it is very difficult to purify a homogeneous population of EVs that is derived from one EV class and from a single cell type that is devoid of co-isolated contaminants such as protein aggregates and lipid-rich particles. Many of the studies referred to in this review article describe purified “exosome” samples which, in reality, are mixtures of microparticles from heterogeneous origins. The same holds true for studies on microvesicles and other microparticles. Given the difficulty in isolating one class of EVs (for example, exosomes) without co-isolating another class (such as microvesicles) due to their overlapping size, density and other physical characteristics, the International Society of Extracellular Vesicles (ISEV) in 2018 published a position statement endorsing the use of the generic term “EV” over the use of any specific EV term when referring to lipid bi-layer-enclosed particles naturally released from cells that do not contain a functional nucleus [6]. In keeping with the ISEV guidelines, this review uses the term “EV” unless otherwise stated by the original source publication. However, the reader should keep in mind that, despite its name/label, all EV samples studied to date are heterogeneous mixtures of microparticles containing multiple EV classes with co-isolated protein and lipid contaminants. Depending on the method of isolation, the microparticle sample may be enriched for a specific EV class and/or cellular origin.

## Extracellular vesicles in neuroinflammation and neurodegenerative diseases

Different cells release different EVs which play different roles at different times depending on the physiologic state. Owing to their biological characteristics, exosomes/EVs can spread around in the CNS passing from one cell to another, bringing along signaling molecules [7]. In health, the immune system and CNS EVs contribute to myelination, trophic support, synaptic plasticity, and antigen presentation of neurons [[8], [9], [10]]. They can also cross the blood brain barrier (BBB) diffusing into peripheral blood to reach other organs, and vice versa with peripheral EVs crossing the BBB to reach the CNS [11]. In disease, EVs in the CNS (whether originating from CNS cells or those that have crossed the BBB from the periphery) can carry oxidative, inflammatory, amyloidogenic, and other damaging molecules that contribute to neuroinflammation and neurodegeneration [11].

## Multiple sclerosis pathogenesis

**Fig. 2:**
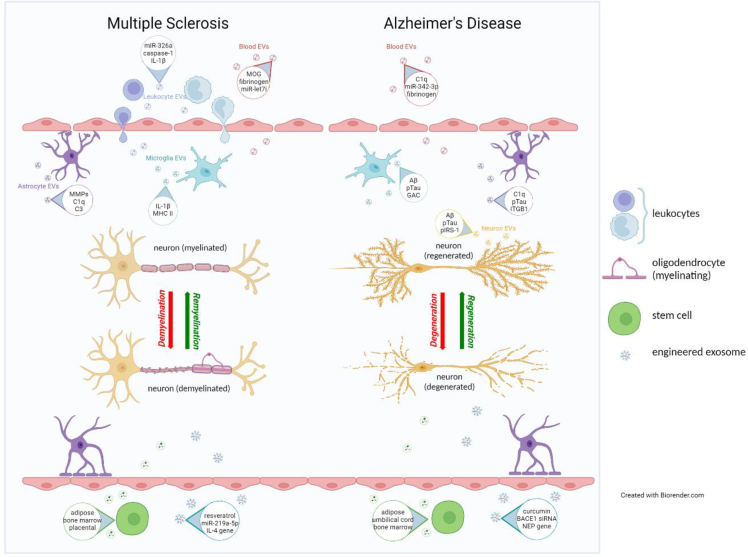
**EV involvement in multiple sclerosis and Alzheimer's disease.** Different cells release different EVs which play varying roles depending on the physiologic state of the secreting cell. During disease, EVs carry biomolecules that facilitate neuroinflammation, neurodegeneration and disrupt the BBB. In health, EVs carry biomolecules that help maintain tissue homeostasis and neurovasculature integrity. Because EVs cross the BBB, EVs derived from MSCs or those specifically engineered to encapsulate therapeutic cargo can enter the CNS to help remyelinate and regenerate neurons.

Multiple sclerosis is a multifactorial, demyelinating disease of the CNS. It is the most common disabling neurological disease of young adults with symptom onset generally occurring between the ages of 20–40 years. It is more common in females than males (3:1 ratio). The disease is characterized by immune system activation, BBB disruption, focal lymphocytic infiltrates, breakdown of myelin sheaths, astrogliosis, microglia activation, and neuronal degeneration. There are four types of MS, listed in order of generally increasing disability or disease severity: clinically isolated syndrome (CIS), relapsing-remitting MS (RRMS), secondary progressive MS (SPMS), and primary progressive MS (PPMS). Each type follows a different disease course and can be further categorized into active or inactive disease depending on whether the condition is progressing at the time. The pathologies seen in MS are a result of multiple inflammatory processes taking place in the periphery, BBB and CNS parenchyma involving a variety of cells --- including immune, endothelial, and CNS cells. All of these cells interact to facilitate the development of MS with EVs participating in some of these interactions [Fig. 2].

An early event in the development of MS lesions is peripheral immune activation and the disruption of the BBB. Primed peripheral lymphocytes mount a reaction against CNS epitopes, including oligodendrocyte proteins such as myelin basic protein (MBP), proteolipid protein (PLP) and myelin oligodendrocyte glycoprotein (MOG). Once activated, these peripheral immune cells (lymphocytes and other leukocytes) release a cascade of cytokines that damage the BBB, allowing leukocyte transmigration and entry into the CNS. While in the CNS, the inflammatory cytokines produced by the leukocytes damage the myelin sheath and injure their parent cells, the oligodendrocytes. In reaction to the damage, reactive gliosis (also known as glial scarring) occurs whereby oligodendrocyte precursor cells, stem cells, microglia and astrocytes are recruited to the site of demyelination where they undergo cellular changes, including activation and cell division. These cellular changes, in coordination with one another, can ultimately lead to the formation of the final glial scar, which consists of a meshwork of tightly interwoven astrocyte processes surrounded by extracellular matrix. A consequence of reactive gliosis and formation of glial scar is failure to regenerate and remyelinate the axons, leading to neurodegeneration.

## Extracellular vesicles in MS pathogenesis

Accumulating evidence implicates EVs released by immune cells, endothelial cells and CNS cells in multiple steps of the MS disease process [Table 2]. With regard to autoreactive T cells, it was shown that miR-326 can induce autoreactive Th17 cell differentiation [12]. This, together with a later study that found increased expression of miR-326 in exosomes derived from conventional T cells of patients with RRMS [13] suggests that T cell exosomes and their miR-326 cargo may promote autoreactivity in MS disease.

**Table 2:** Multiple sclerosis studies with EVs (selected publications).

	EV Source	EV Component	Effect/Finding	Reference
*Pathogenesis*	Blood T cell exosomes	miR-326a	Autoreactivity: ↑ Th17 differentiation	[12,13]
	Blood B cells	Exosomes from B cells of MS patients	↓ Myelination: Induce oligodendrocyte death	[19]
	Plasma Endothelial cells	Plasma CD31+ microparticles (<1.5 mm size)	BBB disruption: ↑ # of plasma microparticles during exacerbation *vs* remission phase and HC, which positively correlated to gadolinium-enhancing lesions seen on MRI (an indicator of BBB disruption)	[14]
	Endothelial cells (TNFα-treated)	CD54+ brain endothelial cell microparticles	BBB disruption & transmigration: 1) *In vitro* studies showed binding, activation and ↑ transmigration of monocytes. 2) ↑ endothelial microparticles in exacerbation *vs* remission phase of MS.	[15]
	Plasma Endothelial cells	Microparticles (<1.5 mm in size)	BBB disruption & transmigration: Plasma from RRMS patients in exacerbation ↑ # of endothelial microparticles and ↑ monocyte transmigration *vs* plasma from remission patients and HC.	[16]
	Plasma Platelets & Endothelial Cells	Plasma microparticles (<3 mm size)	BBB disruption: 1) Plasma EVs derived from platelets and endothelial cells were ↑ in all forms of MS (CIS, RRMS, SPMS, PPMS) *vs* HC. 2) ↑ permeability of endothelial monolayer.	[47]
	CSF Myeloid Cells (Microglia/Macrophage)	CSF microvesicles	↑ inflammation: 1) ↑ Myeloid microvesicle concentration in CSF of EAE mice, CIS and relapsing RRMS patients. 2) Myeloid microvesicles spread inflammatory signals *in vitro* and *in vivo*.	[27]
	Plasma exosomes	miR-let-7i	↑ inflammation: Suppressed induction of Treg cells by targeting insulin like growth factor 1 receptor and transforming growth factor beta receptor 1.	[86]
	Astrocytic EVs	Complement proteins (C1q, C3, C3b/iC3b, C4, C5, C5a, Factor H)	↑ synaptic loss: Increased complement proteins in CSF astrocytic EVs of MS *vs* HC.	[87]
	Plasma EVs	Fibrinogen	Demyelination: 1) Mouse plasma EVs containing fibrinogen injected into EAE mice induced CD8+ T cells and spontaneous relapse. 2) Fibrinogen was also found in human plasma EVs from MS patients.	[21]
	Serum exosomes	MOG	Autoreactivity: 1) ↑ serum exosomal MOG which correlated with gadolinium+ brain lesions. 2) Exosomes from RRMS and SPMS induced ↑ MOG-TCR T cell proliferation vs HC.	[88]
*Biomarkers*	Plasma T cells, B cells, and monocytes	Plasma EVs	Plasma EVs from these cell types were ↑ in untreated RRMS *vs* HC	[48]
	Plasma endothelial EVs (0.2–1 mm)		Diagnose active MS: ↑ # of plasma endothelial EVs in active RRMS *vs* HC and RRMS stable disease	[46]
	Serum oligodendrocyte EVs	MBP	Diagnose and differentiate different MS types: Serum oligodendrocyte EVs from MS patients (CIS, RRMS, PPMS) had ↑ MBP concentration compared to HC, which correlated with disease activity.	[51]
	CSF myeloid EVs	CSF myeloid EVs (≤1 mm in size)	Differentiate between progressive MS and CIS: 1) ↑ # of CSF EVs in progressive MS *vs* CIS. 2) Lesions correlated with ↑ CSF EVs from memory T cells, Th1 cells and Th2 cells.	[49]
	Serum exosomes	Epstein-Barr viral proteins (EBNA1, LMP1, LMP2A)	Diagnose RRMS: 1) ↑ Epstein-Barr viral proteins in RRMS *vs* HC. 2) EBV+ exosomes induced inflammatory response in monocyte-derived macrophages.	[52]
	Serum exosomes	miR-15b-5p, miR-451a, miR-30b-5p, miR-342-3p	Diagnose RRMS: These miRs were ↑ regulated in RRMS *vs* HC.	[50]
	Serum exosomes	miR-127-3p, miR-370-3p, miR-409-3p, miR-432-5p	Diagnose SPMS: These miRs were ↑ regulated in SPMS *vs* HC.	[50]
	Serum exosomes	miR-15b-5p, miR-23a-3p, miR-223-3p, miR-374a-5p, miR-30b-5p, miR-433-3p, miR-485-3p, miR-342-3p, miR-432-5p	Differentiate between RRMS and SPMS: These miRs were ↑ regulated in RRMS *vs* SPMS.	[50]
	Serum Exosomes	miR-122-5p, miR-196b-5p, miR-301a-3p, miR-532-5p	Diagnose RRMS: These miRs were ↓ regulated in RRMS *vs* HC.	[89]

	EV Source	Target	Effect/Finding	Reference
*Therapeutics*	Human adipose MSCs	↓ glial fibrillary acidic protein and Iba-1 in brain. ↑MBP.	Improved motor deficits, ↓ brain atrophy and ↑ remyelination in Theiler's murine encephalomyelitis virus-induced demyelinating disease, a mouse model of MS	[90]
	Human bone marrow MSCs stimulated by IFN*γ*	↓ proinflammatory Th1 and Th17 cytokines (IL-6, IL-12p-70, IL-17AF, IL-22). ↑ immunosuppressive cytokine indoleamine 2,3 dioxygenase	Improved clinical score, ↓ demyelination, ↓ neuroinflammation, and ↑ # of Treg cells in EAE mice.	[91]
	Rat bone marrow MSCs	Regulate microglia polarization	↓ neural behavioral scores, inflammatory cell infiltration, neuroinflammation (↑ IL-10 and TGF-β and ↓ TNF-α and IL-12), and demyelination in EAE rat model.	[92]
	Human placental MSCs	↓ DNA damage in oligodendrocytes	Myelin regeneration: *In vivo* improvement in motor function and ↑ myelination in spinal cord. *In vitro* induction of OPC differentiation into mature myelinating oligodendrocytes.	[93]
	Human umbilical cord MSCs	↓ pro-inflammatory cytokines (IL-17a, TNF-α, and IFN-γ), ↑ anti-inflammatory cytokines (IL-4, IL-10)	Attenuate EAE: Improve clinical score, ↓ pro-inflammatory cytokines (IL-17a, TNF-α, and IFN-γ), ↑ anti-inflammatory cytokines (IL-4, IL-10), ↓ leukocyte infiltration in EAE mice.	[94]
	Mouse oligodendrocyte EVs containing MBP, MOG and PLP	Induce immunosuppressive monocytes and apoptosis of autoreactive CD4+ T cells.	↓ EAE pathophysiology in several EAE models by restoring immune tolerance thru 1) induction of immunosuppressive monocytes, and 2) apoptosis of CD4^+^ T cells.	[95]
	Engineered mouse microglial EVs containing IL-4 plasmid	↑ anti-inflammatory mRNA markers (ym1, arg1) in phagocytic cells.	↓ neuroinflammation and EAE pathophysiology.	[79]
	Engineered HEK 293T cell EVs overexpressing miR-219a-5p	Induce OPC differentiation	Myelin regeneration and clinical score improvement in EAE mice.	[80]
	Engineered EL-4 mouse lymphoma cell-derived exosomes exogenously loaded with curcumin	Anti-inflammation	↓ MOG-induced EAE pathophysiology: Exosome encapsulated curcumin inhibits LPS-induced brain inflammation and myelin oligodendrocyte glycoprotein (MOG) induced autoimmune responses in an EAE model.	[76]
	Engineered mouse macrophage RAW264.7 cell-derived exosomes exogenously loaded with resveratrol (RSV-Exo)	Anti-inflammation (reduce microglia and NF-κB activation)	↓ EAE pathophysiology: In mouse EAE model of MS, intranasal administration of RSV-Exo 1) ↓ inflammatory cytokines (TGF-β, IFN-γ, IL-1β, IL-6, IL-17) and ↑ anti-inflammatory cytokine IL-10 in the brain, spinal cord, spleen and blood. 2) ↓ Iba-1 and NF-κB expression in the brain, spinal cord and spleen. 3) ↓ inflammatory cell infiltration into the brain and spinal cord. 4) ↓ demyelination in the spinal cord.	[78]

Abbreviations: arg1:arginase-1;CIS:clinically isolated syndrome;EAE:experimental autoimmune encephalomyelitis;HC:healthy control;Iba-1:ionized calcium binding adaptor molecule 1;MBP:myelin basic protein;MOG:myelin oligodendrocyte glycoprotein;PLP:proteolipid protein;PPMS:primary progressive multiple sclerosis;RRMS:relapsing remitting multiple sclerosis;SPMS:secondary progressive multiple sclerosis;ym1:chitinase 3-like 3.

The majority of MS lesions occur near disrupted blood vessels and EVs may play a role here by increasing BBB permeability and allowing leukocyte transmigration into the CNS. In a series of experiments by one research group, higher concentrations of microparticles/EVs in the plasma of MS patients in exacerbation versus (*vs*) those in remission or *vs* healthy controls (HCs) were found, with the plasma microparticle concentration correlating to gadolinium-enhancing lesions seen on MRI, an indicator of BBB disruption [14]. Later, it was identified that the population of plasma microparticles that were increased in MS were derived from endothelial cells and *in vitro* experiments demonstrated that endothelial cells activated with tumor necrosis factor-alpha (TNF-α) released microparticles that bound, activated and enhanced the transmigration of monocytes across an endothelial cell layer [15]. A year later, the *in vivo* significance was demonstrated when this group published that plasma from RRMS patients in exacerbation increased monocyte transmigration *vs* plasma from RRMS patients in remission or control plasma, and this transmigration was further increased when the monocytes were treated with microparticles derived from TNF-α-treated endothelial cells [16]. This series of studies implicate endothelial cell EVs in the BBB disruption and the resultant monocyte transmigration pathologies seen in MS disease. Fueling the increased BBB permeability brought on by the endothelial EVs, caspase-1 contained in monocyte microvesicles can injure vascular smooth muscle cells [17], thereby degrading the neurovascular unit and furthering entry of immune cells into the CNS. In addition to monocytes and T cells, the disruption of the BBB may allow entry of B cells [18] that release exosomes capable of inducing oligodendrocyte death, leading to the reduced myelination seen in MS lesions [19]. The disruption in the BBB not only allows infiltration of immune cells but also entry of specific blood factors encapsulated in EVs into the CNS that can promote lesion formation. For example, injection of the blood coagulation protein fibrinogen into mice with disrupted BBB stimulated the recruitment and activation of myelin antigen-specific Th1 cells and peripheral monocytes, leading to encephalitogenic adaptive immune responses and demyelination [20]. Recently, it was found that mouse plasma EVs containing fibrinogen can induce CD8+ T cells and spontaneous relapse when injected into mice with experimental autoimmune encephalomyelitis (EAE), a mouse model for MS [21].

After gaining entry into the CNS, the activated immune cells can release a cascade of inflammatory cytokines into the extracellular milieu that activate CNS cells. The activated CNS cells in turn release proinflammatory cytokines, chemokines, reactive oxygen species and other toxic molecules that mediate neuroinflammation-induced neurodegeneration [22]. One important mechanism of tissue injury in MS is the oxidative damage mediated by activated macrophages and microglia that results in mitochondrial injury responsible for many of the essential pathological features of MS lesions: demyelination, oligodendrocyte apoptosis, axonal degeneration, and structural and functional disturbances of astrocytes and oligodendrocyte progenitor cells [23].

In addition to the above deleterious molecules, activated CNS cells can release EVs to potentiate the neuroinflammation and neurodegeneration seen in MS. For example, reactive astrocytes can secrete microvesicles containing MMPs [24] to further disrupt the BBB and fuel neuroinflammation. Activated macrophages that have trafficked into the brain as well as resident microglia can release extracellular vesicles that contain IL-1β [25], major histocompatibility complex class II (MHC II) molecules [26], and other cargo that further spread inflammatory signals [27]. Indeed, it has been found that myeloid microvesicle concentrations were increased in the cerebral spinal fluids (CSF) of not only relapsing and remitting EAE mice, but also in CIS and relapsing RRMS patients [27].

These studies provide just a sampling of the variety of ways by which EVs can participate in MS pathogenesis. For a more in-depth discussion, the reader is directed to another recent review [28].

## Alzheimer's disease pathogenesis

Alzheimer's disease is the most common neurodegenerative disease manifesting in dementia and is typically a disease of old age. AD is characterized by progressive impairment in cognition, mental state, and ability to carry out daily life activities. The main histopathological features of AD are the accumulation of extracellular neuritic plaques and intracellular neurofibrillary tangles (NFTs), which lead to neuronal cell death and secondary reactive neuroinflammation. AD plaques are spherical microscopic lesions consisting of a core of amyloid beta (Aβ)-peptide surrounded by enlarged axonal endings. Aβ peptides are derived from the proteolytic cleavage of amyloid precursor protein (APP) by a family of enzymes (γ-secretases and β-secretases) that include presenilin 1 and presenilin 2. They are typically 36–43 amino acids long and can self-assemble into oligomers and fibrils that are toxic to neurons. The predominant Aβ fragments found in amyloid deposits are Aβ40 and Aβ42, which have 40 and 42 residues, respectively. Although cleavage of APP by β- and γ-secretases produce pathologic Aβ fragments, cleavage of APP by α-secretase produces soluble peptides that are non-amyloidogenic, neuroprotective and neurotrophic. Multiple mechanisms of toxicity have been attributed to Aβ oligomers, including activation of inflammation, induction of oxidative stress, disruption of membrane receptor functions, formation of membrane pores and alteration of ionic homeostasis across the membrane, and modification of DNA structure [29]. NFTs are formed by aggregates of hyperphosphorylated, tubulin-associated unit (pTau) proteins and can be seen as fibrillary, cytoplasmic structures inside neurons. NFTs cause neuronal dysfunction and death by destabilizing the microtubule network thereby affecting axonal transport. In addition to Aβ plaques and NFTs, synaptic damage and loss are significant features of AD that best correlate with cognitive decline [30]. It is thought that misfolded Aβ and Tau proteins facilitate AD pathology by acting as templates on which healthy proteins misfold and grow into increasingly larger aggregates, and spreading in a prion-like manner [31]. In addition, aggregation of pTau and Aβ activates microglia and astrocytes, triggering an innate immune response that spreads across the brain, leading to the release of inflammatory molecules that contribute to AD progression and severity [32].

## Extracellular vesicles in AD pathogenesis

Although the etiology of AD is unknown, one leading hypothesis is an imbalance between neuronal production of Aβ and extracellular clearance that leads to protein misfolding, aggregation and accumulation in plaques. Evidence indicates that EVs participate in this balance as players on both sides: degradation and formation of amyloid plaques. As early as 2002, Aβ peptides were found in MVBs of normal neurons and more so in AD neurons [33]. In 2006, it was shown that Aβ was secreted extracellularly by neurons in association with exosomes, and the exosomal marker ALIX was enriched around neuritic plaques of AD brains [34]. Then in 2014, it was found that astrocyte exosomes, when co-incubated with Aβ42, induced Aβ aggregation and blocked uptake by glial cells *in vitro*. Moreover, inhibition of exosome secretion by neutral sphingomyelinase 2 inhibitor GW4869 resulted in lower Aβ42 levels and plaque burden in 5XFAD mouse brains, a murine model for AD [35]. In this same year, another group reported that when exposed to Aβ, microglia released microvesicles that were toxic to neurons *in vitro* [36]. In addition, microvesicles isolated from the CSF of AD patients showed increased Aβ bound to neurites and increased neuronal cell toxicity. These data suggest that pathologic Aβ can be encapsulated in exosomes and released by various CNS cells into the extracellular milieu to form plaque.

In contrast to these observations for EVs' role in extracellular Aβ accumulation and neuronal toxicity, there is also evidence supporting their role in amyloid degradation and neuronal health. For instance, it was shown *in vitro* that not only can mouse neuronal exosomes (derived from N2a neuroblastoma cells and from primary cortical neurons) bind to soluble amyloid peptides to prevent their toxic aggregation but mouse N2a neuroblastoma exosomes also facilitated Aβ internalization into microglia for degradation [37]. Later, this same group showed that these neuronal exosomes carried glycosphingolipids essential for Aβ binding and injection of these neuronal exosomes into the brains of APP transgenic mice (a mouse model for AD) reduced Aβ and amyloid depositions [38,39]. By sequestering Aβ, not only can neuronal exosomes deliver this toxic material to microglia for degradation, but they can also neutralize Aβ′s toxic effects on synaptic plasticity [40]. It is unclear whether the seemingly opposing roles of EVs are due to limitations in EV sample purity and reproducibility across laboratories, or a reflection of the variable nature of EVs whose cargo changes dynamically to reflect the temporal and physiologic state of the secreting cell.

In addition to Aβ, exosomes/EVs from different cell types have been shown to contain and affect phosphorylated Tau protein homeostasis. For example, when compared to cognitively normal subjects, plasma neuronal EV levels of pTau-181 and pTau-396 were not only higher in the AD patients but when these neuronal EVs were injected into wildtype mouse brains, greater pathologic pTau protein levels were observed, suggesting that neuronal EVs can seed and propagate Tau pathology onto normal brains [41]. For microglial EVs, Asai and colleagues showed that they can also carry and spread Tau [42]. Specifically, when treated with human Tau oligomers, cultured primary mouse microglial cells phagocytosed and secreted the Tau proteins in exosomes. In addition, deleting microglia from mouse brains led to reduced Tau propagation from the entorhinal cortex to the dentate gyrus regions of the brain. Finally, *in vitro* inhibition of specific exosome secretion by microglia resulted in reduced Tau transmission to neurons while *in vivo* general inhibition of exosome secretion led to reduced pTau in mouse brains. For astroglial EVs, it was shown that when exposed to Aβ, human cortical astrocytes produced more of the pTau species associated with NFTs and released them within exosomes [43]. More recently, an *in vivo* study of AD brains shows the presence of pTau species in astrocytes of the dentate gyrus which correlated with Braak stage severity, and overexpression of the 3R Tau isoform in mouse dentate gyrus astrocytes led to neuronal disruptions and spatial memory impairment [44]. Interestingly, the 3R Tau inclusions seen in this study were not only higher in AD individuals but exacerbated in those with amyloid plaques. Together, these studies suggest that the loss of Tau homeostasis in astrocytes can contribute to AD-like symptoms and astrocytic EVs containing pTau may contribute to that imbalance. Further implicating and, perhaps, emphasizing the role of EVs in Tau spread, it was observed that neuronal uptake of Tau *in vivo* was shown to be higher when Tau is encapsulated in exosomes *vs* its naked form when injected into mouse brains [42].

Much of the work on EVs in AD has focused on their role as vehicles for Aβ and Tau spread. However, EVs have also been shown to participate in other cellular mechanisms found disrupted in AD, such as impaired lysosome degradation, neuroinflammation, BBB disruption, synaptic dysfunction, and diminished neuroprotection [Fig. 2] [Table 3]. For information on EV roles in these other mechanisms, see the recent review by Gomes and colleagues [45].

**Table 3:** Alzheimer's disease studies with EVs (selected publications).

	EV Source	EV Component	Effect/Finding	Reference
*Pathogenesis*	HeLa (human epithelial), N2a (mouse neuroblastoma) exosomes	Aβ	Aβ propagation: 1) These cells release exosomes containing Aβ. 2) ALIX was enriched around neuritic plaques of AD brains.	[34]
	Neuronal exosomes from AD brain	Aβ	Aβ propagation between neurons and neuronal toxicity: 1) Exosomes from AD brains have increased Aβ. 2) These exosomes were internalized by neuronal cultures, resulting in cytotoxicity.	[54]
	Plasma neuronal EVs	pTau-181, pTau-396, Aβ42	pTau seeding and propagation: 1) pTau-181, pTau-396 and Aβ42 were all higher in AD. Neurogranin and REST were lower in AD. 2) Mice brains injected with plasma nEVs from AD patients showed ↑ pTau in brain tissue.	[41]
	Plasma neuronal EVs	ratio of P-serine-IRS-1 to P-tyrosine-IRS-1	Insulin resistance: 1) ↑ P-serine-IRS-1 and ↓ P-tyrosine-IRS-1 in AD *vs* matched controls. 2) These changes in phosphorylated IRS-1 were seen in preclinical AD and plateaued as early as 10 years before clinical diagnosis of manifest AD.	[64]
	Microglial microvesicles	Aβ40, Aβ42	Neuronal toxicity: 1) Aβ42-treated microglia released microvesicles which contained Aβ42 and Aβ40. 2) CSF myeloid microvesicle number was ↑ in AD *vs* HC. 3) Neurons incubated with microvesicles derived from CSF of AD patients showed increased Aβ bound to dendrites and increased neuronal cell death.	[36]
	Mouse microglial EVs	Aβ42	Aβ propagation between neurons and synaptic dysfunction: Aβ42-treated mouse microglia release EVs containing Aβ42 which 1) alters dendritic spine morphology *in vitro*, and 2) when injected into mouse brains, causes synaptic dysfunction by impairing low-term potentiation in the entorhinal cortex which then spreads to the dentate gyrus.	[57]
	Primary cultured murine microglial exosomes	Tau	Tau spread to neurons: 1) mouse microglia phagocytosed and secreted Tau in exosomes. 2) Mice depleted of microglia had 47% and 70% reductions in pTau in the entorhinal cortex and dentate gyrus, respectively.	[42]
	Mouse microglial EVs	pTau	Tau propagation: 1) Microglia depletion in mouse brains ↓ pTau propagation, and ↑ plaque burden and plaque-associated pTau^+^ dystrophic neurites. 2) Neurodegenerative/Mac2^+^ microglia, released >3 times more EVs than homeostatic/Mac2^-^ microglia in humanized APP mutant knock-in (*App*^*NL-G-F*^) mice.	[56]
	Microglial EVs from early AD mouse brain	glutaminase C (GAC)	Neuroinflammation thru microglial activation: 1) GAC protein level is higher in APP/PS1 mouse brains *vs* control mice. 2) *In vitro* plasmid-transfected mouse microglia overexpressing GAC shifted to a proinflammatory state and released more exosomes containing proinflammatory miRs (miR-155, miR-130, miR-145a, miR-23b, miR-146a & others).	[60]
	Primary human cortical astrocytic exosomes	Tau and pTau	Tau propagation: Aβ-treated astrocytes have increased intracellular pTau which are secreted in association with exosomes.	[43]
	Plasma astrocytic EVs	IL-6, TNF-α, IL-1β, complement proteins (C1q, C4b, C3d, factor B, factor D, Bb, C3b, MAC)	Neuroinflammation and neuronal damage in late AD: 1) IL-6, TNF-α, IL-1β and complement effector proteins (C1q, C4b, C3d, factor B, factor D, Bb, C3b, MAC) were ↑ in AD *vs* cognitively normal controls while complement regulatory proteins (CD59, CD46, decay-accelerating factor (DAF) and complement receptor type 1 (CR1)) were ↓. 2) Complement protein levels correlated with severity of disease.	[58]
	Astrocytic EVs from AD brains	aquaporin 4 (AQP4)	BBB disruption: 1) Injection of astrocytic EVs isolated from postmortem familial AD & sporadic AD brains into mice induced perivascular astrocyte reactivity and reduced blood vessel integrity. 2) 3xTg-AD mice astrocytes released more EVs *vs* control mice.	[62]
	AD brain-derived EVs	Tau oligomers	pTau seeding and propagation: 1) Alzheimer's disease extracellular vesicles had ↑ pathologic/PHF1^+^ pTau and were more efﬁciently internalized by murine cortical neurons. 2) These AD-EVs were more capable of misfolding and transferring Tau *in vitro vs* prodromal AD/MCI and control EVs. 3) Inoculation of AD brain-derived EVs resulted in the accumulation of abnormally phosphorylated Tau throughout the hippocampus	[96]
*Biomarkers*	Plasma neuronal EVs from AD patients	pTau-181, pTau-396, Aβ42	Preclinical diagnosis: 1) pTau-181, pTau-396 and Aβ42 were all elevated in AD. 2) These neuronal EV biomarkers were also elevated 10 years before symptom onset and at the time of diagnosis.	[53]
	Plasma neuronal EVs from AD patients	Increased levels of pTau (pTau-181, pTau-231), Aβ42 and pIRS-1	Preclinical diagnosis: In a large case-control study, combining these neuronal EV biomarkers predicted AD about 4 years before symptom onset. Also, individual biomarkers were associated with cognitive performance.	[55]
	Plasma neuronal exosomes	cathepsin D, LAMP-1, ubiquitin, Hsp70	Preclinical diagnosis and monitor disease progression: 1) AD patients had higher exosomal levels of cathepsin D, LAMP-1 and ubiquitin proteins, and lower exosomal levels of Hsp70. 2) These 4 protein changes were evident up to 10 years before clinical onset. 3) Compared to FTD, AD had higher cathepsin D, LAMP-1 and ubiquitin.	[61]
	Plasma neuronal exosomes from AD patients	presynaptic proteins (NRXN2a, NLGN1, AMPA4 receptor)	Preclinical diagnosis and monitor disease progression: ↓ presynaptic proteins (NRXN2a, NLGN1, AMPA4 receptor) in preclinical period 6–11 years before the onset of dementia *vs* matched controls, which declined progressively with dementia development. 2) ↓ presynaptic proteins (NLGN1, AMPA4 receptor) in AD *vs* Controls, which inversely correlated with ADAS cognition score.	[71]
	Plasma neuronal exosomes	synaptic proteins (GAP43, neurogranin, SNAP25, and synaptotagmin 1)	Preclinical diagnosis and monitor disease progression: ↓ GAP43, neurogranin, SNAP25, and synaptotagmin 1 in AD *vs* Controls, which correlated with disease severity and MMSE scores (i.e., levels are lowest in AD, then aMCI, then Controls). 2) Combination of these four synaptic proteins can predict AD-associated cognitive impairment 5–7 years before appearance of symptoms (Area Under the Curve = 0.89).	[74]
	CD11b^+^ microglial EVs (<200 nm in size)	FTH1, TREM2, Tau	Diagnose AD: Loss of homeostatic microglia: 1) ↑ Tau and disease associated microglia markers (FTH1 and TREM2) in AD *vs* normal controls. 2) ↓ microglia homeostatic markers (P2RY12, TMEM119) in AD vs normal Controls.	[66]
	AD brain-derived astrocytic EVs	integrin-β1 (ITGB1)	Diagnose AD: ↑ integrin-β1 in AD brain samples, which correlated with AD hallmarks (Aβ42, total Tau and pTau-396)	[67]
	Plasma astrocyte exosomes	complement proteins	Monitor disease progression: ADE levels of complement proteins (C1q, C4b, factor D, fragment Bb, C5b, C3b, and C5b-C9) were ↑ in patients with MCIC (MCI converting to dementia within 3 years) *vs* MCIS (MCI stable). ADE levels of inhibitory complement proteins (decay-accelerating factor, CD46, CD59, and type 1 complement receptor) were ↓ in MCIC *vs* MCIS.	[72]
	Brain fluid-derived EVs	pathological Tau species	Differentiate AD tauopathy from other tauopathies: Brain fluid-derived EVs isolated from AD brain tissues contained Tau which, when injected into THY-tau30 mouse brains, induced Tau phosphorylation and conformational changes in AD *vs* non-demented controls, progressive supranuclear palsy (PSP) or Pick's disease (PiD).	[75]
	CSF exosomes	pTau-181	Diagnose AD: pTau-181 in CSF exosomes are ↑ in AD *vs* non-AD controls.	[69]
	AD plasma exosomes	20 differentially expressed miRs	Diagnose AD: 20 miRs were differentially expressed in AD *vs* matched HC (23b-3p, 24-3p, 29b-3p, 125b-5p, 138-5p, 139-5p, 141-3p, 150-5p, 152-3p, 185-5p, 338-3p, 342-3p, 342-5p, 548 at-5p, 659-5p, 3065-5p, 3613-3p, 3916, 4772-3p, 5001-3p)	[68]
	AD platelet-poor plasma EVs	MOG, miRs (Let-7g-5p, 126-3p, 142-3p, 146a-5p, 223-3p)	Monitor disease progression: 1) ↑ MOG in AD *vs* controls, which correlated with disease severity/dementia level as assessed by MMSE score. 2) ↓ miRs (Let-7g-5p, 126-3p, 142-3p, 146a-5p, 223-3p) in AD *vs* Controls, which inversely correlated with disease severity/dementia level as assessed by MMSE score.	[73]
	EVs from postmortem AD blood	fibrinogen proteins (FGG, FGA, FGB), complement C6, peroxiredoxin 2, phosphatidylethanolamine-binding protein 1, peroxiredoxin 2, carboxypeptidase N subunit 2, pigment epithelium-derived factor, fatty acid binding protein 1, serine protease inhibitor 10	Distinguish sporadic AD from controls: 1) Blood microvesicles (100–1000 nm) are increased in AD vs controls. 2) fibrinogen proteins (FGG, FGA, FGB), complement C6, peroxiredoxin 2, phosphatidylethanolamine-binding protein 1, peroxiredoxin 2, carboxypeptidase N subunit 2, pigment epithelium-derived factor, fatty acid binding protein 1 and serine protease inhibitor 10 distinguished sporadic AD from control.	[63]

	EV Source	Target	Effect/Finding	Reference
*Therapeutics*	Human adipose-derived MSC EVs	neuroprotective and neurogenic genes and proteins	Ameliorate neurologic damage and increase neurogenesis in APP/PS1 mouse model for AD: Decreased Aβ deposition, reduced microglia activation, rescue memory deficits.	[97]
	Rat bone marrow-derived MSC EVs (BM-MSC-EVs) containing miR-29c-3p	BACE1	Ameliorate AD pathology: 1) AD rats injected intracerebrally with BM-MSC-EVs containing miR-29c-3p had ↓ cognitive impairment (as assessed by the Morris water maze test), ↓ Aβ deposition, ↓ inflammatory cytokines (IL-1β, IL-6, TNF-α), and ↑ Aβ-degrading enzymes (NEP, IDE). 2) BM-MSC-EVs containing miR-29c-3p blocked BACE1 expression and activated Wnt/β-catenin pathway.	[98]
	Human bone marrow-derived MSC (hBM-MSC) small EVs	Aβ and astrocyte activation	Ameliorate AD pathology: 5XFAD AD mice given hBM-MSC EVs intranasally showed improved cognition, ↓ Aβ plaque load in hippocampus, and ↓ colocalization of GFAP and Thioflavin S, suggesting reduction of astrocyte activation surrounding plaques.	[84]
	Wharton's jelly (human umbilical cord connective tissue) MSC exosomes	Aβ, synaptic plasticity genes (BdnfIV, SYP, GluR, GRIN2B)	Improve cognitive and memory deficits and ameliorate AD pathology: Reduced Aβ plaque burden, inhibited astrocyte activation, and upregulated memory- and synapse-related genes in AD transgenic mouse model.	[99]
	Human neural stem cell (hNSC)-derived EVs	targets of miRs (125b-5p, 124-3p, 125a-5p)	Ameliorate AD pathology: 5xFAD transgenic AD mice intravenously injected with hNSC-EVs had improved cognition (assessed by behavioral testing), ↓ synaptic loss (↑ synaptophysin expression), ↓ Aβ plaque, and ↓ microglial activation/CD68 expression. 2) hNSC-EVs contained miR-125b-5p, miR-124-3p and miR-125a-5p.	[100]
	Engineered mouse bone marrow-derived dendritic cell exosomes containing BACE1 siRNA	BACE1, Aβ42	Reduce BACE1 mRNA and protein in wildtype mouse brains: Intravenous injection of exosomes from immature murine dendritic cells loaded with siRNA for BACE1, a protein implicated in AD, led to targeted reduction of BACE1 and Aβ42 proteins in cortical tissue.	[81]
	Engineered human umbilical cord-derived MSC (UMSC) EVs containing neprilysin (NEP) gene	Aβ cleavage	Reduce Aβ plaque burden, increase neurogenesis and promote anti-inflammation: Compared to Aβ42-injected AD mice with unmodified UMSCs, mice injected with *NEP* gene-containing UMSCs had EVs in brains that 1) contained more NEP and less BACE1 proteins, 2) had fewer neuritic plaques, and 3) showed greater neurogenic and anti-inflammatory properties (↑ BDNF, ↑ NeuN, ↓ GFAP, ↓ Iba-1) in hippocampus.	[82]
	Engineered mouse macrophage RAW264.7 cell exosomes exogenously loaded with curcumin	block Tau phosphorylation by activating AKT/GSK-3β pathway	Reduce neuronal cell death and cognitive impairment in AD mouse model (okadaic acid injection into mouse brains): Reduced okadaic acid-induced neuronal cell death in hippocampus and reduced cognitive impairment as measured by the Morris water maze test.	[77]

Abbreviations: aMCI:amnestic mild cognitive impairment;APP:amyloid precursor protein;BACE1:beta-site amyloid precursor protein cleaving enzyme type 1 (β-secretase 1);BDNF:brain-derived neurotrophic factor;FTH1:ferritin heavy chain-1;GFAP:glial fibrillary acidic protein;HSF-1:heat shock factor 1;Iba-1:ionized calcium-binding adaptor molecule 1;IDE:insulin degrading enzyme;IRS:insulin receptor substrate;LAMP 1:lysosome-associated membrane protein 1;MAC:membrane attack complex;MMSE:Mini Mental State Exam;MOG:myelin oligodendrocyte glycoprotein;MSCs:mesenchymal stem cells;NeuN:neuronal nuclear protein;NEP:neprilysin;P-serine-IRS-1:phospho-serine-type I insulin receptor substrate;P-tyrosine-IRS-1:phospho-tyrosine-type I insulin receptor substrate;PAR-4,prostate apoptosis response 4;PHF1:pSer396/pSer404 Tau;pIRS-1:phosphorylated insulin receptor substrate 1;PS1:presenilin 1;P2RY12:purinergic receptor P2Y12;REST:repressor element 1-silencing transcription factor;TMEM119:transmembrane protein 119;TREM2: triggering receptor expressed on myeloid cells 2.

## EV biomarkers in MS and AD

Access to pathologically relevant tissues is extremely difficult for neurological diseases. Because EVs are released from disease-relevant cells into accessible biofluids and they can cross the BBB-carrying cell- and disease-specific molecular signatures, they are an attractive source for biomarkers in diagnosing, monitoring and treatment of disease. As a form of “liquid biopsy”, EVs may be preferable over biofluids because they not only offer a protective shell for possibly sensitive biomolecules, but there is also a lower probability of contaminants and irrelevant molecules being present. Furthermore, EVs immunopurified from specific cell types may have an even lower contaminant burden and higher relevant cargo. Most of the biomolecules put forth as potential biomarkers for MS and AD are miRs and proteins that have been found in EVs isolated from the blood and CSF [Table 2, Table 3]. The source of EVs can originate from a variety of cell types. Although it is frequently the case that an increase in EV number is associated with disease, it is often not known the precise cargo of the EVs and how the various biomolecules they contain affect disease.

For MS, the quantity of EVs in the plasma and CSF has been shown to be increased in MS patients compared to HCs as well as increased cell type-specific EVs have been identified [14,27,[46], [47], [48]]. Furthermore, EV numbers may be increased during the exacerbation *vs* remission phase of the disease [15,46,49]. In some instances, the EV cargo was interrogated and some biomarkers were identified which can differentiate one clinical form of disease from another. For example, a set of nine miRs (miR-15b-5p, miR-23a-3p, miR-223-3p, miR-374a-5p, miR-30b-5p, miR-433-3p, miR-485-3p, miR-342-3p, miR-432-5p) were identified in serum exosomes that were upregulated in RRMS and distinguished it from SPMS with 80% accuracy [50]. Myelin basic protein (MBP) in serum oligodendrocyte EVs was recently identified as being upregulated in MS patients *vs* HCs with MBP levels being highest in PPMS, followed by RRMS, and then CIS [51]. Because Epstein-Barr virus (EBV) is a risk factor for AD, EBV proteins in serum exosomes from MS patients have also been investigated with results showing not only are the EBV proteins EBNA1, LMP1 and LMP2A increased in RRMS *vs* HCs, but their levels are higher in active *vs* stable disease [52].

For AD biomarkers, much of the focus has centered on finding Aβ and Tau, the components of the characteristic hallmarks of AD, in EVs isolated from blood or CSF that are derived from different cell types. Neurons [34,41,[53], [54], [55]], astrocytes [43] and microglia [36,42,56] have all been shown to release EVs containing Aβ or Tau that participate in AD neurodegeneration. In addition to Aβ and Tau, other cargo has been identified in EVs released by CNS cells that participate in AD-associated pathologies such as synaptic impairment [57], inflammation [[58], [59], [60]], lysosomal dysfunction [61], BBB disruption [62,63], and insulin resistance [55,64]. Due to overlapping pathomechanisms, sometimes the same molecular EV cargo has been identified in both AD and MS as being potential biomarkers (e.g., c1q, fibrinogen, miR-342-3p). Like MS, the number of exosomes/EVs released in AD may be higher during pathologic conditions [56,62,63]. This may be a result of endolysosomal dysfunction that pushes unwanted molecules towards the endocytic pathway where they are disposed of in the form of exosomes [65]. In addition to the detection of disease [[66], [67], [68], [69]], specific EV cargo has also been identified that can distinguish between different types of AD [63] as well as monitor the progression of disease [61,[70], [71], [72], [73], [74]]. Moreover, the detection of preclinical AD as early as 10 years prior to clinical diagnosis of manifest AD has been reported using plasma neuronal EV biomarkers [64], and frontotemporal dementia may be differentiated from AD by differences in plasma neuronal EV cargo [70]. Because changes in Tau are associated with other neurological diseases, it has also been suggested that EVs may serve as biomarkers of AD-specific tauopathy since EVs found in brain fluids of AD patients carry different pathological Tau species that distinguish them from other tauopathies, such as Pick's disease and progressive supranuclear palsy, which display morphologically different types of Tau aggregates [75].

## EV therapeutics in MS and AD

Being natural products of the body, exosomes/EVs are less immunogenic. Along with their ability to transport cargo across the BBB, it is no wonder that exosomes/EVs are being explored as possible therapeutic vehicles. One therapeutic approach stems from the observation that EV release is increased during pathology. Thus, blocking EV secretion is one possible avenue. Indeed, it was shown that intraperitoneal injection of the pharmacologic drug GW4869, which targets neutral sphingomyelinase 2, into 5XFAD mouse brains decreased levels of brain exosomes, ceramides and Aβ42 plaque [35]. Another approach is through the harvesting of EVs from *in vitro* cell cultures of mesenchymal stem cells (MSCs) which contain therapeutic cargo that attenuate disease through a variety of mechanisms including reducing inflammation, inducing myelination, decreasing Aβ42, blocking Tau phosphorylation, and augmenting synaptic plasticity [Table 2, Table 3]. A third approach is the modification of naive EVs through exogenous loading of therapeutic agent by co-incubation, sonication, electroporation or transient permeabilization. Examples of exogenous cargo loaded into EVs which have shown promise as therapeutics for MS and AD include curcumin [76,77], resveratrol [78], plasmid expressing IL-4 anti-inflammatory cytokine [79], lentiviral particles containing miR-219a-5p [80], β-secretase (BACE1) siRNA [81], and plasmid expressing neprilysin, an enzyme which cleaves Aβ [82].

Since EVs can cross the BBB, they can be administered by intravenous, intraperitoneal, subcutaneous or intranasal delivery to treat CNS diseases. In addition, studies have shown they can be successfully administered both systemically and locally to provide a therapeutic effect at a desired location [83]. To improve the targeted delivery of exosomes for the treatment of CNS diseases, a specific ligand/receptor or antibody/antigen binding strategy is often used to promote the coupling of exosomes to target cells, thereby facilitating internalization. For AD, intranasal administration has shown promise in animal models [84] and there is currently a human clinical trial registered to test the efficacy of allogeneic adipose tissue-derived MSC exosomes administered by nasal drip (NCT04388982). Due to their potential as biomarkers, treatment and drug delivery vehicles, clinical trials of EVs have accelerated in the past 3 years, in large part due to interest and funding for COVID-19 disease. Despite this recent growth, the EV therapeutic field is still at a very early stage. Most studies are in early phases without a single completed phase III clinical trial.

## Limitations, closing remarks and future prospective

Despite the explosion of interest and research in the past decade, the EV field is still in its infancy and its application to CNS diseases is rapidly growing. Evidence to date suggests a very promising potential for EVs to be biomarkers and therapeutics for MS and AD. Nevertheless, some intellectual and technical barriers must be overcome before EVs can be applied to patients. Among the intellectual barriers is the insufficient knowledge about which cells may be the most useful for producing EVs and how best to load EVs with therapeutic molecules. On the technical side, EV isolation in high yield and high purity while preserving their intact structure and biological activity is a very big challenge currently. Moreover, due to the heterogeneity of EVs, standardization in isolation and characterization is sorely needed to enable comparison between laboratories to more accurately assess EV function. The lack of standardization may be one reason why reproducibility across laboratories, thus far, is poor with sometimes conflicting results being reported. With technological advances continuing to be developed, it is the hope that someday the power of EVs can be harnessed to facilitate targeted drug delivery to specific CNS cells to combat neuroinflammation and neurodegeneration to ameliorate MS, AD and other CNS diseases.

## Conflicts of interest

The author declares no financial or personal conflicts of interest. Due to space limitations, not all original research findings were included and references to more generalized information were excluded.
